# Mucinous Cystadenoma Arising in Post-Treatment Non-Seminomatous Germ Cell Tumor: A Rare Occurrence

**DOI:** 10.7759/cureus.74375

**Published:** 2024-11-24

**Authors:** Harshit Agrawal, Vikas K Panwar, Ankur Mittal, Siddharth Saxena, Ashok Singh

**Affiliations:** 1 Urology, All India Institute of Medical Sciences, Rishikesh, Rishikesh, IND; 2 Pathology, All India Institute of Medical Sciences, Rishikesh, Rishikesh, IND

**Keywords:** mucinous tumours, retroperitoneal lymph node dissection, rplnd, testicular germ cell tumor, yolk-sac tumor

## Abstract

Germ cell tumors, the most common of the testicular neoplasms, originate from primordial germ cells. These tumors are known for their totipotent nature, capable of differentiating into various cell types. This case report presents a rare occurrence of mucinous cystadenoma in a patient who received chemotherapy for metastatic left non-seminomatous germ cell tumor (NSGCT) of the testis. Despite undergoing chemotherapy, a residual retroperitoneal mass persisted. The persisting large mass is usually suggestive of teratoma or malignancy. Upon surgical removal, the histopathology revealed a surprising diagnosis: that of mucinous cystadenoma.

## Introduction

Germ cell tumors (GCTs) are the most common testicular neoplasms which have their origin in primordial germ cells. These tumors are known for their totipotent nature, capable of differentiating into various cell types. GCTs are broadly classified as germ cell neoplasia in situ (GCNIS)-derived and non-GCNIS-derived. The majority of post-pubertal GCTs are GCNIS-derived. For clinical purposes, GCNIS-derived GCTs are divided into seminoma and non-seminomatous germ cell tumor (NSGCT), and the relative distribution of each is 52%-56% and 44%-48%, respectively [1]. Following first-line BEP (Bleomycin, Etoposide, Cisplatin) treatment, it has been reported that about 7% of residual masses in NSGCTs contain active cancer, 33% post-pubertal teratoma, and 40% necrotic-fibrotic tissue only [2]. Occasionally these residual masses can present with novel histopathology.

## Case presentation

A 37-year-old male patient presented with complaints of dull aching abdominal pain for the past three months. The patient was diagnosed with carcinoma of the testes three years back. Initially, three years back, he noticed a mass in his left testicle, which was evaluated and managed by a local healthcare facility. Ultrasound of the scrotum revealed a hetero-echoic testicular mass suggestive of carcinoma testis. The patient underwent a trans-scrotal orchidectomy as the initial surgical intervention. Histopathological examination of the excised testis revealed a non-seminomatous germ cell tumor (NSGCT). The initial histopathology was yolk sac tumor with associated extensive granulation tissue and necrosis.

Subsequent to the diagnosis, the patient underwent hemi-scrotetomy and high inguinal cord excision. Following the surgery, the patient received four cycles of BEP chemotherapy, which included the agents Bleomycin, Etoposide, and Cisplatin. Despite being advised of a high risk of recurrence, which requires a vigilant follow-up, the patient was lost to follow-up post-chemotherapy.

Over the ensuing three years, the patient experienced no major health concerns until the onset of vague, dull abdominal pain that gradually progressed in intensity over the past three months. Following these complaints, the patient presented to the Urology Department of the All India Institute of Medical Sciences (AIIMS), Rishikesh, and was evaluated.

On presentation, the patient denied any associated symptoms such as fever, weight loss, hematuria, or bowel or urinary disturbances. There was no history of recent trauma, infection, or any new mass development in the scrotal region.

Given his oncological history, the clinical suspicion of a recurrence or metastasis of the testicular tumor was raised. The patient was subjected to a comprehensive evaluation, including a repeat assessment of serum tumor markers and a contrast-enhanced CT (CECT). These included alpha-fetoprotein (AFP), beta-human chorionic gonadotropin (B-HCG), and lactate dehydrogenase (LDH). All his serum markers were within normal limits.

**Figure 1:**
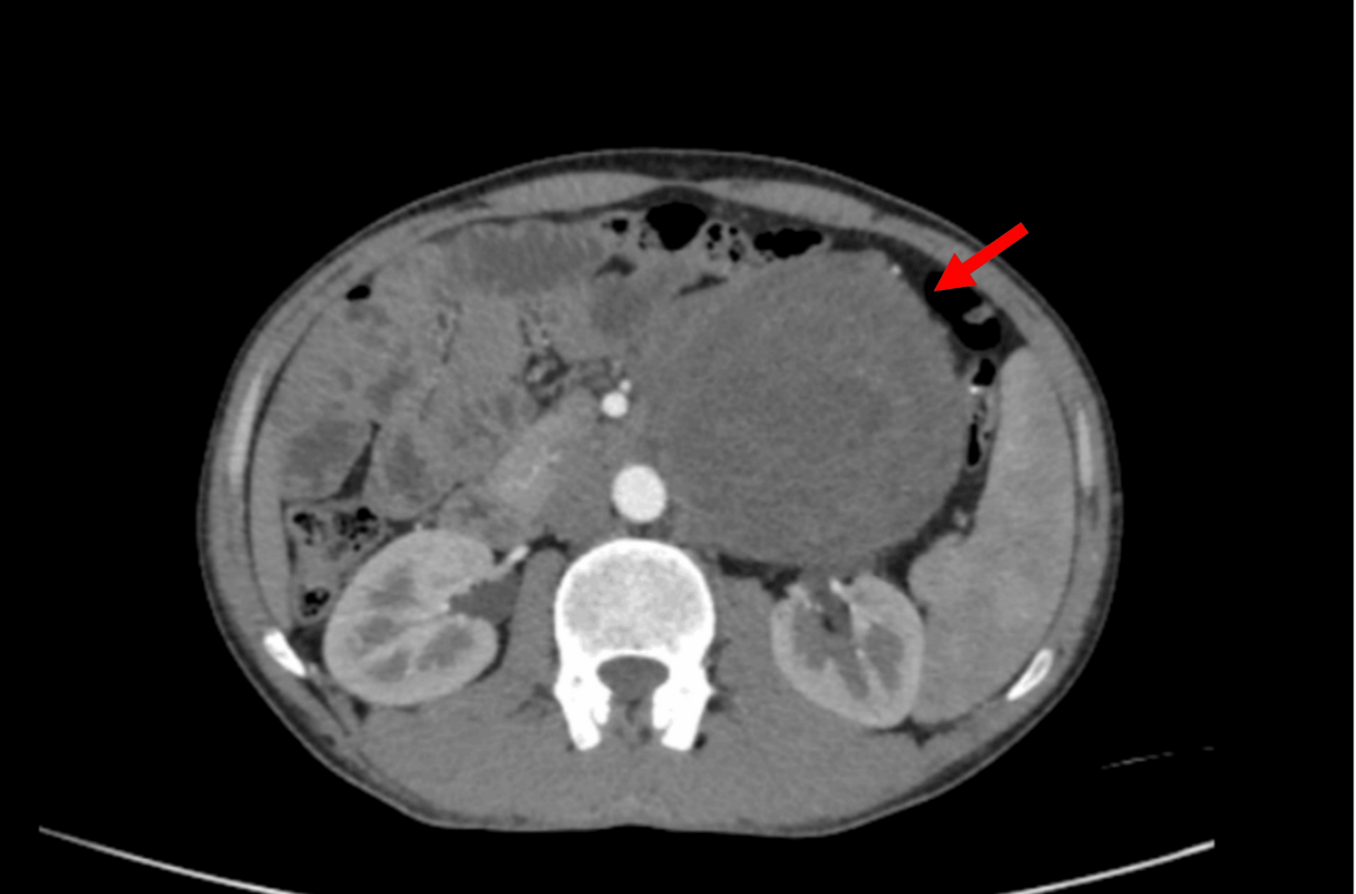
CT image (axial) The red arrow indicates the residual tumor post-chemotherapy

**Figure 2:**
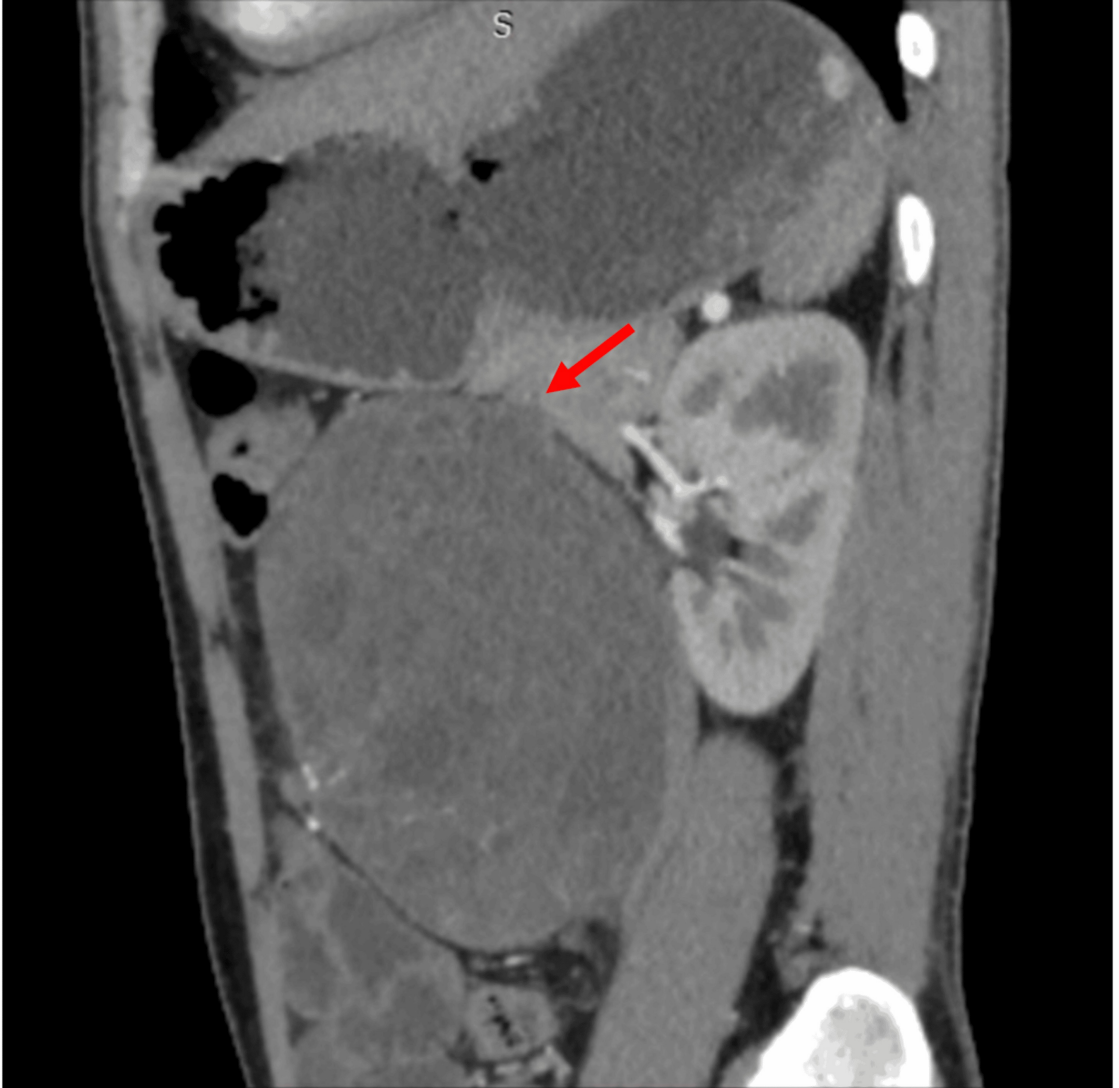
CT image (sagittal) The red arrow indicates the post-chemotherapy residual lesion

The CT findings revealed ill-defined heterogeneously enhancing mass lesion of size 11.3x11.4x14 cm, noted in the left lumbar region with internal necrotic areas. Posteriorly, it was infiltrating into the left psoas muscle. Postero-superiorly, it was abutting the pancreatic tail. It was abutting left renal vessels with loss of fat planes. Laterally, it was abutting the descending colon with focal loss of fat planes. Anteriorly, it was reaching up to the recti and abdominal wall muscles with maintained fat planes. Medially, it is abutting and displacing the descending aorta and spinal muscular atrophy (SMA) with maintained contrast opacification (Figures 1, 2).

**Figure 3:**
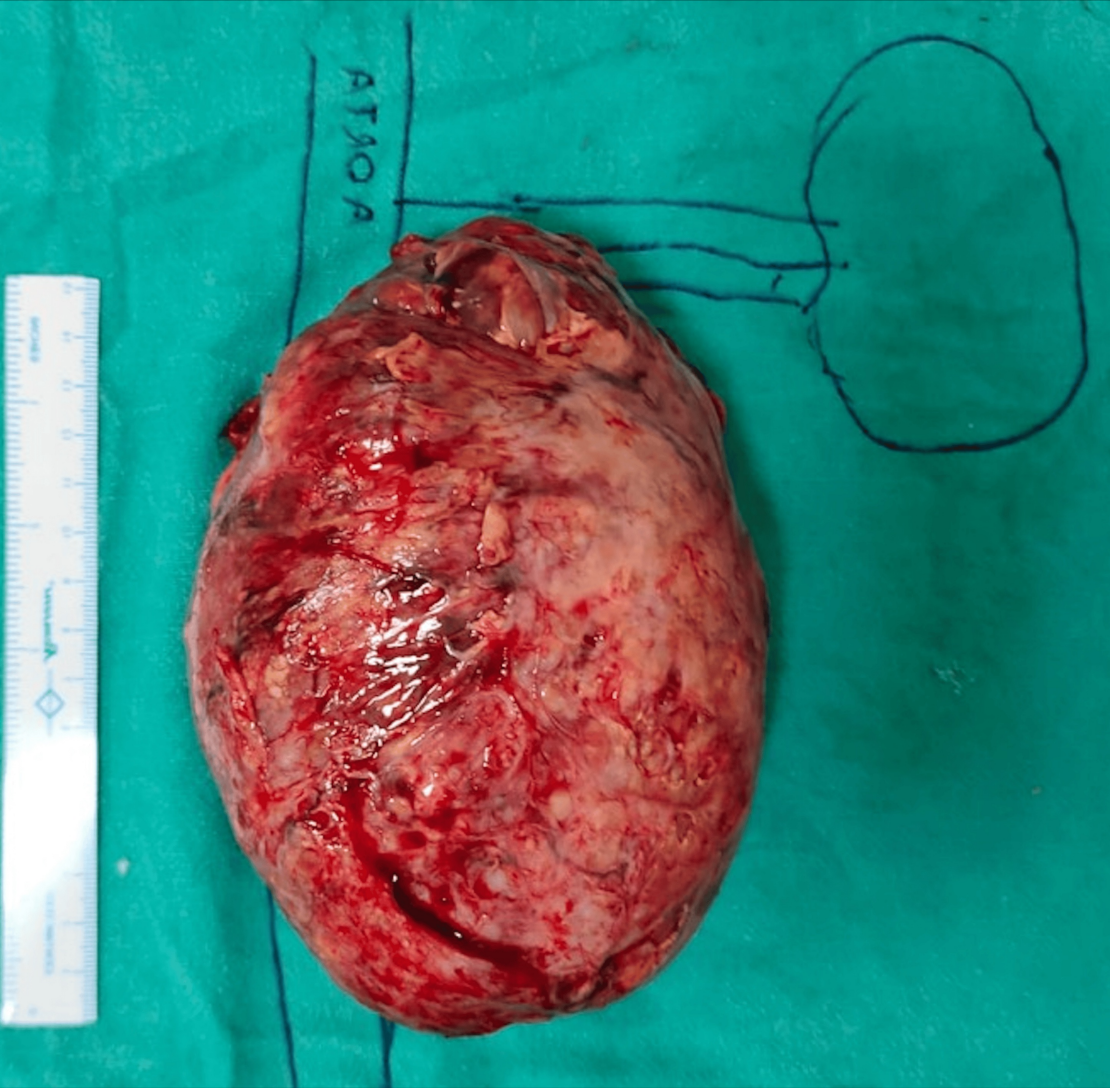
Surgical specimen after resection A large 17 cm x 14 cm x 8 cm mass resected from retroperitoneum

The patient underwent open retroperitoneal mass excision with left-template retroperitoneal lymph-node dissection (RPLND). The lesion had dense adhesions present between mass and psoas muscle and aorta, and a part of psoas was also excised and repaired. The excised lesion had the dimensions of 17 cm x 14 cm x 8 cm (Figure 3).

Post-op recovery was uneventful, and the patient was discharged on post-operative day 3. The patient is doing well at three-month follow-up with normal serum markers and no recurrent lesion.

**Figure 4:**
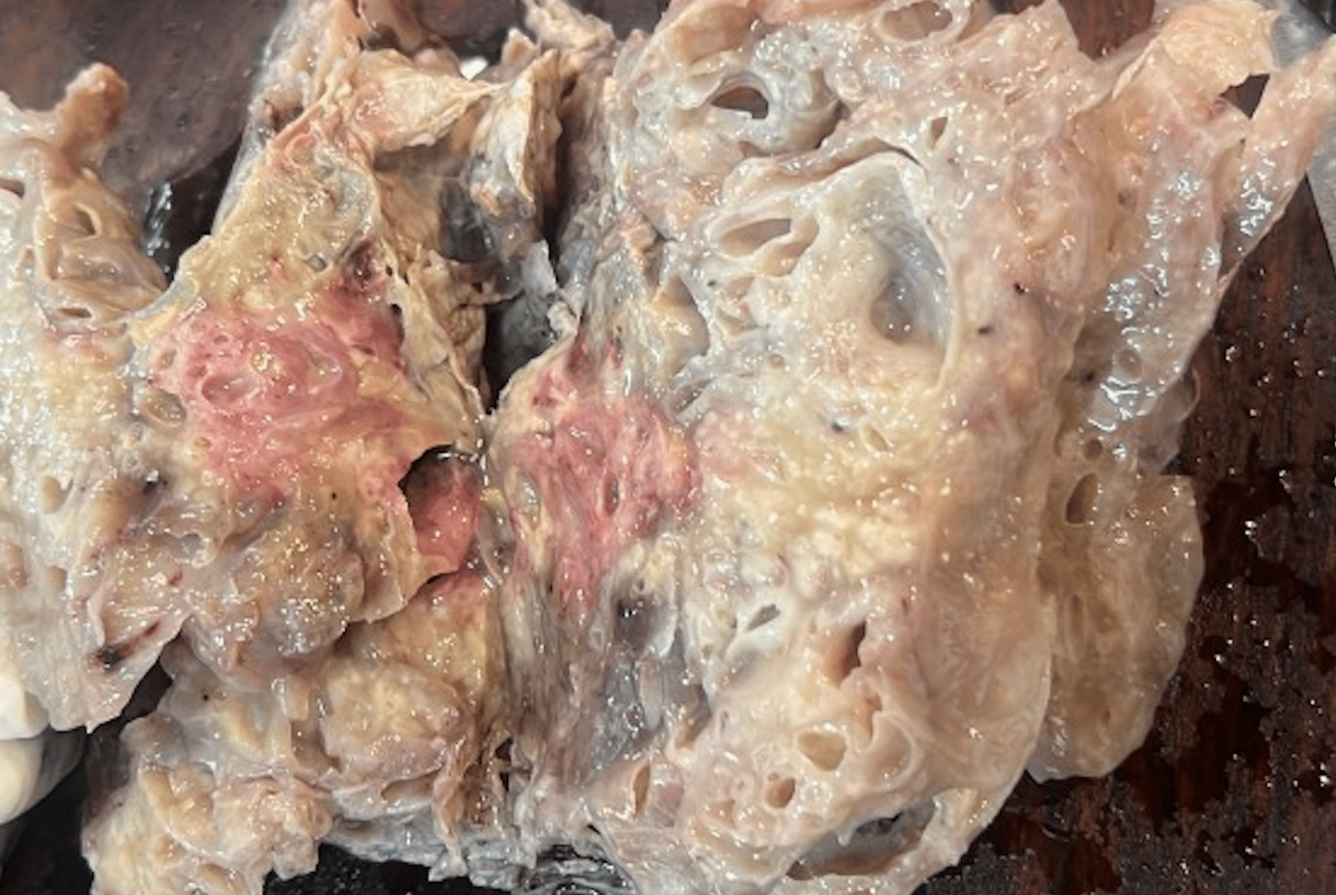
Cut Open Specimen The lesion consists of multiple cystic cavities filled with mucinous content

**Figure 5:**
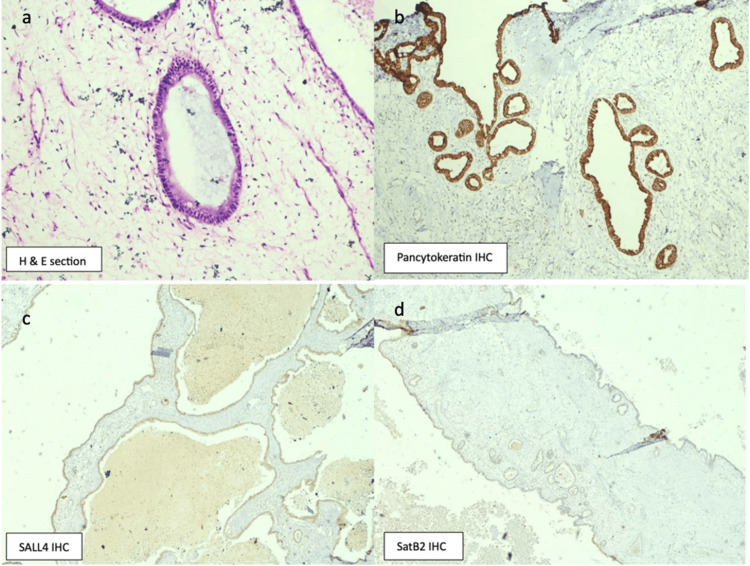
Histopathology (a) Hematoxylin and eosin (H and E) section (100x) shows a cyst lined by columnar epithelial cells with the presence of  mucin; (b) pan-cytokeratin is strong and diffuse membranous positive; (c) SALL4 is negative; (d) SatB2 is negative

The histopathology showed a multi-loculated cystic mass with diffuse fibro-collagenous capsule. The lumen of the cyst was filled with mucinous proteinaceous material (Figure 4). The histopathology showed a tumor made up of large cystic spaces lined by columnar epithelial cells with the presence of mucin. No cellular atypia, multilayering or invasion was seen (Figure 5). Immunohistochemistry (IHC) markers for p40, SATB2, SALL4, and PANCK were placed. They were positive for only PANCK. The IHC thus confirmed mucinous cystadenoma.

## Discussion

Retroperitoneal lymph node dissection (RPLND) for post-chemotherapy nodal masses of more than 1 cm is a standard of care in metastatic non-seminomatous germ cell tumor (NSGCT). In multiple studies, including by Conduit et al. [2], the most common histopathology reported for post-chemotherapy RPLND is necrosis/fibrosis in 40% of the patients, with a relative minority of patients having teratoma (33%) or viable tumor (7%) reported within the specimen.

Yolk sac tumors are relatively uncommon tumors seen rarely in the adult population. A study by Goldberg et al. showed a similar low incidence of yolk sac tumor [3]. There is no study available that is focused on post-chemotherapy RPLND histopathology.

Only one case was reported in the English literature by Chahal et al. [4], which showed a simultaneous existing mucinous cystadenoma and non-seminomatous germ cell tumor. The mucinous cystadenoma was of urachal origin and not part of RPLND. Mucinous cystadenoma is frequently seen in tumors of the ovarian origin and occasionally seen in tumors of pancreatic origin. Mucinous cystadenoma accounts for about 21% of ovarian tumors and second most common benign tumor of the ovary [5]. To our knowledge, this is the first case reporting mucinous cystadenoma following RPLND in post-chemotherapy metastatic NSGCT.

## Conclusions

Germ cell tumors are characterized by their capacity for totipotent differentiation. Mucinous cystadenoma most commonly originates from ovarian tumors as surface epithelial tumors. They are occasionally of pancreatic origin. Both these tumors can occasionally be associated with malignancy. Mucinous cystadenoma after RPLND in post-chemotherapy retroperitoneal mass in a case of non-seminomatous germ cell tumor is previously unreported, to our knowledge, and this is the first instance as such, thereby making it a possible diagnosis in NSGCT patients. It can be considered a possible diagnosis when large cystic masses are seen on imaging and when the cyst is lined by columnar epithelial cells with the presence of mucin in histopathology.

## References

[1] McGlynn KA, Devesa SS, Sigurdson AJ, Brown LM, Tsao L, Tarone RE (2003). Trends in the incidence of testicular germ cell tumors in the United States. Cancer.

[2] Conduit C, Hong W, Martin F (2022). A meta-analysis of clinicopathologic features that predict necrosis or fibrosis at post-chemotherapy retroperitoneal lymph node dissection in individuals receiving treatment for non-seminoma germ cell tumours. Front Oncol.

[3] Goldberg H, Klaassen Z, Chandrasekar T, Fleshner N, Hamilton RJ, Jewett MA (2019). Germ cell testicular tumors - contemporary diagnosis, staging and management of localized and advanced disease. Urology.

[4] Chahal D, Martens M, Kinahan J (2015). Mucinous cystic tumour of low malignant potential presenting in a patient with prior non-seminatous germ cell tumour. Can Urol Assoc J.

[5] Batool A, Rathore Z, Jahangir F, Javeed S, Nasir S, Chughtai AS (2022). Histopathological spectrum of ovarian neoplasms: a single-center study. Cureus.

